# Ethylene Response Factor *Sl-ERF.B.3* Is Responsive to Abiotic Stresses and Mediates Salt and Cold Stress Response Regulation in Tomato

**DOI:** 10.1155/2014/167681

**Published:** 2014-08-06

**Authors:** Imen Klay, Julien Pirrello, Leila Riahi, Anne Bernadac, Ameur Cherif, Mondher Bouzayen, Sadok Bouzid

**Affiliations:** ^1^Laboratoire de Morphogenèse et de Biotechnologie Végétale, Faculté des Sciences de Tunis (FST), Campus Universitaire 2092 El Manar Tunis, Tunisia; ^2^Université Toulouse, INP ENSA Toulouse, 31326 Castanet-Tolosan, France; ^3^INRA, 31326 Castanet-Tolosan, France; ^4^LR Biotechnologie et Valorisation des Bio-Géo Ressources (LR11ES31), Institut Supérieur de Biotechnologie, Université de La Manouba Biotech Pole de Sidi Thabet, Sidi Thabet, 2020 Ariana, Tunisia

## Abstract

*Sl-ERF.B.3 (Solanum lycopersicum ethylene response factor B.3)* gene encodes for a tomato transcription factor of the ERF (ethylene responsive factor) family. Our results of real-time RT-PCR showed that *Sl-ERF.B.3* is an abiotic stress responsive gene, which is induced by cold, heat, and flooding, but downregulated by salinity and drought. To get more insight into the role of *Sl-ERF.B.3* in plant response to separate salinity and cold, a comparative study between wild type and two *Sl-ERF.B.3* antisense transgenic tomato lines was achieved. Compared with wild type, *Sl-ERF.B.3* antisense transgenic plants exhibited a salt stress dependent growth inhibition. This inhibition was significantly enhanced in shoots but reduced in roots, leading to an increased root to shoot ratio. Furthermore, the cold stress essay clearly revealed that introducing antisense *Sl-ERF.B.3* in transgenic tomato plants reduces their cell injury and enhances their tolerance against 14 d of cold stress. All these results suggest that *Sl-ERF.B.3* gene is involved in plant response to abiotic stresses and may play a role in the layout of stress symptoms under cold stress and in growth regulation under salinity.

## 1. Introduction

Plants are frequently exposed to a plethora of stress conditions such as low temperature, salt, drought, flooding, heat, oxidative stress, and heavy metal toxicity. Abiotic stress often leads to plant growth inhibition and limits crop productivity [1]. It has been estimated that abiotic stresses were the principal cause of decreasing the average yield of major crops by more than 50% [2]. To confront such environmental aggressions, plants develop adaptive responses at physiological and molecular levels, which are specified to each stress condition [3].

Indeed, plants activate a number of defense mechanisms that function to increase tolerance to the adverse conditions imposed by stresses. A major event in response to stresses is the perception and transduction of stress signals through signaling components, which results in the activation of numerous stress-related genes [4]. The products of these genes may participate in the generation of regulatory molecules like the plant hormones ethylene, abscisic acid (ABA), and salicylic acid (SA). These regulatory molecules can, in turn, initiate a second round of signaling that contribute in the final plant response to these abiotic stresses [5].

One of the most important regulatory molecules that are related to environmental responses in plant species is ethylene. As a gaseous plant hormone, ethylene is proved to be involved in plant stress responses, in addition to its roles in germination, fruit ripening, organ abscission, pathogen response and senescence, and so forth [6]. Several reports suggested that accumulation of ethylene or its precursor, the ACC (aminocyclopropane-1-carboxylic acid) is exceedingly induced by abiotic stress stimuli such as salinity [7], water stress [8], and flooding [9].

Ethylene function is exerted through modulation of gene expression that was operated in part at transcriptional level by ERF (ethylene response factor) considered as the effectors of ethylene signal. In* Arabidopsis*, a linear ethylene transduction pathway was proposed, which corresponds to a succession of components from ethylene receptor integrated in the endoplasmic reticulum [10] to transcription factors localized in the nucleus [11].

ERF transcription factors, a huge multigene family of transcription factors regulating the expression of ethylene dependent genes, are the most prominent components directing the specific and diversified plant responses to the ethylene signal. Members of the ERF transcription factor family play important roles in regulating gene expression in response to biotic and abiotic stresses [12]. Ethylene-responsive element-binding factors (ERFs) form a plant-specific transcriptional factor superfamily of 147 members in* Arabidopsis* [13]. Interestingly, depending on the circumstances, ERFs can function as both activator and/or repressor elements [14, 15]. They can also act as an integrative node and common transcription factor of different signalling pathways [16].

ERF proteins are characterized by the presence of highly conserved sequence. This sequence, named ERF domain, provides ERF affinity to the GCC box, comprised in promoter region of ethylene responsive gene. Several studies suggested that ERF proteins have the capacity to specifically bind not only to GCC box, but also to the DRE/CRT motif (found in promoter region of stress responsive genes), known as a* cis*-acting element that responds to cold or osmotic stress [17, 18].

Previous studies reported the involvement of many ERF genes in plant environmental stress responses in many plant species, especially in* Arabidopsis* [19–21]. Tomato (*Solanum lycopersicum*) is one of the most important agricultural crops and increasing knowledge on their ERF genes involved in abiotic stress responses is a real challenge and can help in plant improvement programs. However, the role of tomato ERFs in response to such stresses remains not enough elucidated.

The present study is the first to examine the expression patterns of the transcription factor gene* Sl-ERF.B.3* (formerly called* LeERF4*) in tomato to determine its regulation in response to a variety of abiotic stress conditions (salinity, drought, flooding, heat, and cold) based on wild type tomato plants. Given that expression pattern of a gene under different conditions can unravel its functionality [22], we used real time RT-PCR approach to study the transcript abundance levels of the studied gene during several abiotic stress responses.

We further focused on* Sl-ERF.B.3* role in vegetative growth regulation against salt stress by characterizing two* Sl-ERF.B.3* antisense transgenic tomato lines compared to wild type, and we discussed the* Sl-ERF.B.3* role in the layout of shoot and root growth changes related to adaptive responses to salinity. Moreover, we investigated* Sl-ERF.B.3* antisense lines tolerance against cold treatment and* Sl-ERF.B.3* role on cell membrane injury occurred during low temperature stress responses.

## 2. Materials and Methods

### 2.1. Plant Material

In the first part of this study, variations on the* Sl-ERF.B.3* gene expression under five types of abiotic stresses (salinity, drought, flooding, heat, and cold) were investigated, based on wild type tomato plants (*Solanum lycopersicum* cv. MicroTom). In the second part of this work we focused on the tomato plant responses to salt and cold stresses. For this purpose, in addition to the investigated wild type tomato, two homozygous independent antisense transgenic tomato lines (*Sl-ERF.B.3.AS42* and* Sl-ERF.B.3.AS38*) (provided by the Laboratory of Genomic and Fruit Biotechnology, UMR990 INRA/INP-ENSA Toulouse, France) were included in this part of our study.

### 2.2. The* Sl-ERF.B.3* Gene Expression Study

*Growth Conditions*. Wild type tomato plants were grown in compost (jiffy pots) under growth chamber conditions (16 h light/8 h darkness, 25°C and 80% humidity); all seedlings were allowed to grow for 21 d before stress treatment.

*Abiotic Stress Treatments*. All stress treatments (salinity, drought, flooding, heat, and cold) were applied at the same vegetative growth stage (three-week-old plants). All experiments were repeated three times and each replicate corresponds to six plants. For the drought assay, watering was withheld for 5 d from plants grown in jiffy pots whose weights were beforehand equilibrated by adding appropriate volume of water to maintain the same soil water levels before drought application. The salt stress was achieved by watering with 250 mM NaCl solution and shoots harvesting 24 h later. For the cold stress assay, plants were incubated at 4°C for 8 h. To apply heat stress, plants were exposed to 42°C for 8 h. The flooding stress was applied by immersing plants (at cotyledon level) in deionised water for 72 h. Immediately following application of each type of stress, the aerial parts were removed and frozen in liquid nitrogen for later use for RNA extraction.

*RNA Extraction and cDNA Synthesis*. Total RNA was extracted by using an RNeasy RNA Isolation kit (Qiagen, Valencia, CA, USA). Then the RNA was treated with RNase-free DNase (Qiagen, Valencia, CA, USA) at 25°C for 1 h. After quantification, two micrograms of total RNA were incubated at 65°C for 5 min, then placed for 2 min on ice, and were used for cDNA synthesis. cDNA was synthesized using Omniscript Reverse Transcriptase enzyme (Qiagen, Valencia, CA, USA) in a total volume of 20 *μ*L, incubated at 37°C for 1 h.

*Quantitative Real-Time PCR (qRT-PCR)*. Real-time RT-PCR was carried out to determine the expression levels of* Sl-ERF.B.3* (GenBank accession number AY192370) in wild type line under adverse abiotic stress conditions, by using specific primers. qRT-PCR was also performed for* Sl-actin-51* (*Solanum lycopersicum-Actin-51*), as endogenous control, and for three reference genes:* Dehydrin CI7* [23],* Hsp21* [24], and* ACO1* [25]. The gene-specific primer pairs used for the qRT-PCR are listed in Table 1.

qRT-PCR was performed using cDNA, corresponding to 2 ng of total RNA, in a 10 *μ*L reaction volume using SYBR GREEN PCR Master Mix (PE-Applied Biosystems, Foster City, CA, USA), on an ABI PRISM 7900HT sequence-detection system. qRT-PCR conditions were as follows: 50°C for 2 min, 95°C for 10 min, then 40 cycles of 95°C for 15 s and 60°C for 1 min, and finally one cycle 95°C for 15 s and 60°C for 15 s. For all real-time RT-PCR experiments, each reaction was run in triplicate on 384-well plate. To determine the relative fold difference for each sample in each experiment, we used the comparative 2^−ΔΔCT^ method, where the Ct (threshold cycle) value for* ERF* gene was normalized to the Ct value for* Sl-Actin-51* (*Solanum lycopersicum-Actin-51*) and was calculated relative to a calibrator using the formula 2^−ΔΔCT^ (where fold change = 2^−ΔΔCT^, ΔCt = Ct  (ERF  gene) − Ct (*Sl- Actin-51*), and ΔΔCt = ΔCt (stress) − ΔCt (control)).

### 2.3. Growth Test of Antisense Lines under Salt Stress

Fifty disinfected seeds per line (WT, AS25, and AS38) were placed in Petri dishes on one layer of filter paper, moistened with 15 mL of sterile water, and incubated at 25°C in the dark. Salt treatment was operated by shifting the obtained seedlings into MS (Murashige and Skoog) agar medium supplemented with NaCl (corresponding to 200 mM concentration). Seven days after, seedlings were transplanted in compost (fertilized at regular intervals by nutrient solution), under growth chamber conditions and watered three times a week by 200 mM NaCl solution. Growth parameters (stem height, root length, and fresh weight) were assessed after six weeks of salt stress. A mean of four biological repetitions were made.

### 2.4. Measurement of Electrolyte Leakage in Antisense Lines under Cold Stress

Three-week-old plants from tomato wild type and* Sl-ERF.B.3* antisense transgenic lines, grown, in jiffy pot, under optimal conditions, were transferred at 2°C for 14 days. After cold exposure, the electrolyte leakage was measured on leaves from both cold treated plants and control plants maintained at 25°C. Briefly, eight leaf discs were taken from each plant and incubated in 10 mL of deionized water for three hours under shake at 150 rpm with a horizontal agitator. The electrical conductivities (EC0) of the obtained solutions were determined using a conductivity meter. Then the leaf discs in deionized water were boiled for 15 min. After being thoroughly cooled to room temperature, the conductivities (ECt) of the resulting solutions were determined. The electrolyte leakage was calculated as the ratio of EC0 to ECt. Each data is the average value from three to six independent replicates.

### 2.5. Data Analysis

Pairwise comparisons were made with Student's *t*-test. For gene expression level data, comparison was made between mRNA level in control sample and that in stress treated sample. For growth parameters and electrolyte leakage data, comparison was made between wild type and antisense transgenic plants under abiotic stress and under control conditions.

Sequence alignments were made using ClustalW tool (default parameters) of MEGA software (version 5.0; http://www.megasoftware.net) [26]. Similar sequences search was executed using the BlastP (Protein Basic Local Alignment Search Tool) tool at the NCBI (http://blast.ncbi.nlm.nih.gov/Blast.cgi) web site.

## 3. Results and Discussion

### 3.1. Expression of Tomato* Sl-ERF.B.3* Gene under Different Abiotic Stress Treatments

**Figure 1:**
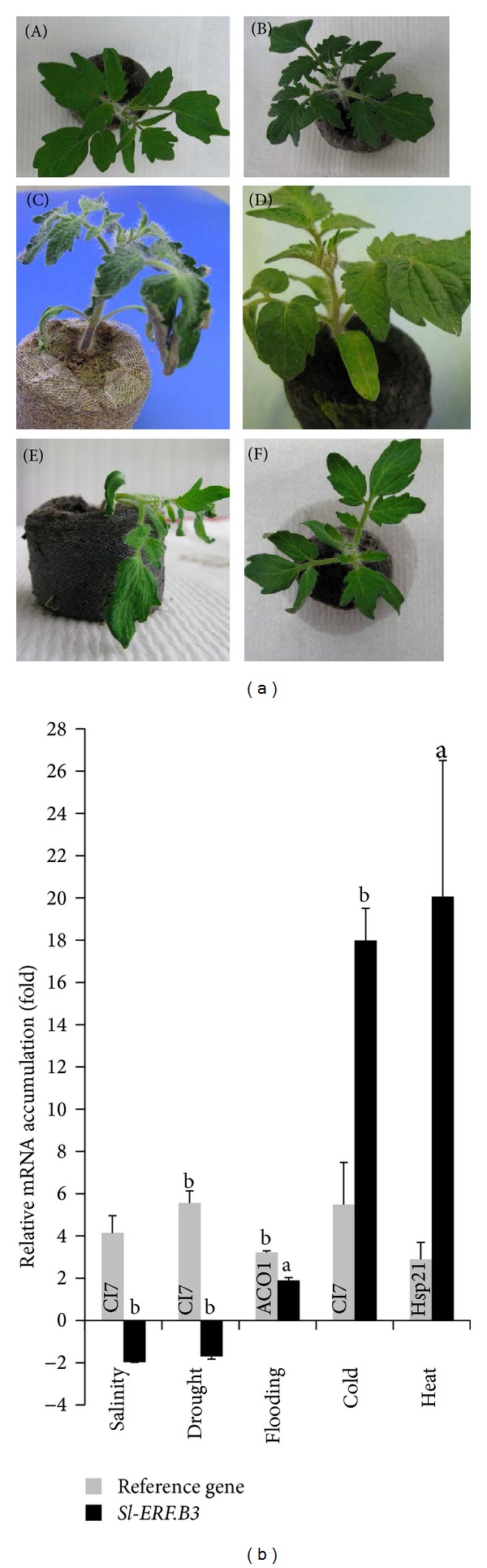
Expression pattern of* Sl-ERF.B.3* gene, in aerial parts of wild type tomato plants, in response to five abiotic stresses. Transcripts accumulation was assessed by quantitative real time PCR, as described in material and methods, using total RNA samples extracted from shoots of three weeks old wild type tomato plants: treated by 4°C for 8 h (cold), stored at 42°C for 8 h (heat), watered by 200 mM NaCl solution during 24 h (salinity), watering deprived during 5 d (drought), or root inundated during 72 h (flooding). (a) Plants used for RNA extraction refer to those stress treated (by salinity (B); by drought (C); by flooding (D); by cold (E); or by heat (F)) and (A) for no stress-control. For each case one representative plant is presented. (b) The stress treatments effect on* Sl-ERF.B.3 *and three reference genes (*CI7, ACO1,* and* Hsp21*) expression level. The* Sl-actin-51* transcripts in the same samples were used as internal control. Data are expressed as relative values, based on the values of control taken as reference sample set to 1.0. Pairwise comparisons were made between control and abiotic stress treated wild-type plants with Student's *t*-test (a: *P* < 0.05, b: *P* < 0.01). Data represent means and standard error of three replications.

In order to decipher the regulation of tomato* Sl-ERF.B.3* gene expression, under the effect of various abiotic stresses, we have performed qRT-PCR approach. This molecular technique allowed us to assess the* Sl-ERF.B.3* expression level in wild type (WT) tomato plants treated or not by five types of environmental stress: cold, heat, flooding, drought, and salinity.  Figure 1(a) shows characteristic phenotypes of plants which were used for RNA extraction. Compared with control, all treated plants displayed various stress symptoms such as wilting, cotyledon yellowing, and leaf curling. The effectiveness of the abiotic stress treatment was confirmed by the accumulation of the stress reference genes transcripts:* Dehydrin CI7* in the salt, drought or cold-treated samples and* Hsp21* and* ACO* in, respectively, heat and flooding treated samples (Figure 1(b)). Relative fold differences in the gene expression levels were calculated by comparing stress treated tomato plants and the corresponding control plants. Our results revealed that* Sl-ERF.B.3* transcripts were highly accumulated in shoots when plants were subjected to heat (20-fold difference), cold (18-fold difference), or flooding (1.9-fold difference). However, the* Sl-ERF.B.3* mRNA accumulation was twofold lesser when plants were exposed to salinity or drought. This suggests that* Sl-ERF.B.3* is really an abiotic-stress-responsive transcription factor, which is upregulated by flooding, cold, or heat, but downregulated by drought or salinity. These environmental stresses contribute to the regulation of* Sl-ERF.B.3* expression. Furthermore, it is evident that physiological plant response is critically affected by environmental conditions, and this response is the result of molecular changes, including mostly transcriptional regulators. Such regulators can be common to several environmental stimuli or specific to one kind of stress factor. This leads to support the possibility that* Sl-ERF.B.3* is a common regulator of the plant response to many abiotic stress conditions including extreme temperatures, drought, high salinity, and flooding and that it can be a key regulator of plant responses to abiotic stress. It is worth noting that* Sl-ERF.B.3/LeERF4* had not previously been reported to be associated with any kind of the predicted abiotic stresses.

The similar trend of regulation of* Sl-ERF.B.3*, under separately high salinity and drought stress conditions (Figure 1(b)) indicates that* Sl-ERF.B.3* seems to be involved in the cross-talk between the plant responses to these stresses. Probably because both salt and drought stresses lead to common constraints (the water deficit and osmotic stress) [27] and can have a similar stress adaptation mechanism. The fact that different stress adaptation mechanisms share the same regulator suggests its prominent regulator role in the complex stress response network. It was previously shown that salt stress leads to disruption of normal metabolism. In tomato, most of the genes of metabolism were downregulated by salt stress, especially genes related to cell wall metabolism [28]. In this respect, it was demonstrated that upon severe salt stress, this decrease of metabolism was due to water limitation that occurred during drought as well as salt stress. The osmotic stress was caused by the decrease of the soil water potential, which conduced to lowering water availability. This dehydration constraint led to various stress injury at the origin of multiple cellular responses, including changes in membrane shape, solute concentration, denaturation of proteins, and production of active oxygen species [29, 30].

Given that our results indicate that cold, heat, and at a lesser extent flooding treatments, all separately trigger* Sl-ERF.B.3* upregulation in tomato shoots; these three stresses should induce similar stress adaptation mechanisms. Although, high and low temperature stresses are apparently two antagonistic environmental constraints encountered by plants, they lead to similar trend of* Sl-ERF.B.3* regulation. This supports the asserting that they have common features and can recruit common molecular response, without being identical [31]. Certainly, at cell level, stress factors such as high and low temperature modify immediately the membrane fluidity by influencing the expression of genes coding plasma membrane proteins [32, 33]. In this context, it was demonstrated in soybean that various plasma membrane proteins were upregulated under the effect of flooding stress, in order to cope with damages of oxygen lack, mostly proteins related to antioxidative system and heat shock cognate protein [34].

### 3.2. Transgenic Plants Growth Response to Salt Stress

**Figure 2:**
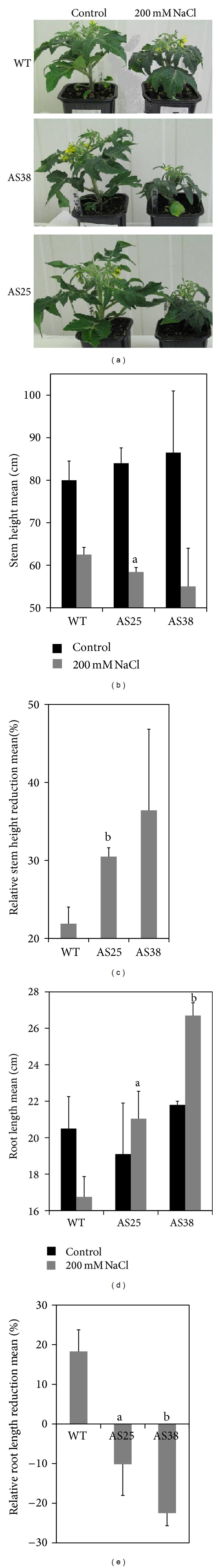
Salt stress effect on wild type and* Sl-ERF.B.3* antisense lines growth. (a) Photographs of both* Sl-ERF.B.3* antisense transgenic tomato lines (AS38 and AS25) and wild type (WT) were taken at sampling time after six weeks of treatment (irrigation by 200 mM NaCl solution). Control: plants were grown under normal growth conditions. Arrows show the leaf epinastic curvature induced by salt stress. Stem height (b) and root length (d) means are assessed in two* Sl-ERF.B.3* antisense transgenic tomato lines (AS25 and AS38) and in wild type (WT) grown for six weeks on compost in absence (black bars) or in presence of NaCl (200 mM NaCl) (grey bars) in irrigation solution. Percentages of relative reduction in stem height mean (c) and that in root length mean (e) between control (0 mM NaCl) and salt stressed (200 mM NaCl) plants of both wild type (WT) and* Sl-ERF.B.3* antisense transgenic lines (AS25 and AS38) are presented. Data are means of three replications ± standard error. Letters indicate significant differences between wild type and transgenic lines according to Student's *t*-test (a: *P* < 0.05, b: *P* < 0.01).

To study the role of* Sl-ERF.B.3* gene in the regulation of tomato vegetative growth under NaCl stress, we evaluated several growth parameters (stem height, root length, and fresh weight) in* Sl-ERF.B.3* antisense transgenic tomato plants and in untransformed WT. Under normal growth conditions, differences in stem growth level were noted between* Sl-ERF.B.3* antisense transgenic plants and WT (Figure 2(a)). These transgenic lines displayed slightly better height growth than the untransformed WT (Figure 2(b)), but statistic analysis revealed no significant differences between values. Consequently, the presence of antisense* Sl-ERF.B.3* in transgenic plants could not have a significant role in their stem elongation, under normal growth conditions. In tomato, epinasty which is induced by ethylene is a good indicator of salt-sensitivity [35], and ethylene biosynthesis is increased by salt stress [7]. Introducing antisense* Sl-ERF.B.3* gene into tomato plants results in less pronounced epinastic phenotype (Figure 2(a)). In transgenic plants, the mRNA of the introduced antisense* Sl-ERF.B.3*, highly likely binds to target sense mRNA and blocks protein synthesis. Further, the two different antisense lines showed similar phenotype suggesting that it is likely due to the downregulation of* Sl-ERF.B.3* gene by introducing its antisense shape. Therefore,* Sl-ERF.B.3*, acting as activator in the ethylene signal transduction pathway, may be involved in the layout of the epinastic leaf curvature phenotype.

After six weeks of salt stress treatment (200 mM NaCl), and as seen in Figures 2(b) and 2(c), both WT and transgenic plants exhibited salt related stem growth decrease, which is clearly higher in* Sl-ERF.B.3* antisense transgenic lines (significant differences between AS25 and WT). In contrast, the salt stress dependent curved leaf aspect is more pronounced in WT plants (Figure 2(a)). Interestingly, compared with that growing at control conditions, the relative decrease in stem expansion, observed in stressed plants (200 mM NaCl) is higher in transgenic lines than in WT (Figure 2(c)). Indeed, in response to 200 mM NaCl, more than 36% of relative stem height reduction was assessed in AS38 transgenic line for only 21.8% in WT plants. Thus, the long time exposure to salt stress leads to a considerable NaCl stress dependent stem growth inhibition in tomato plants (whatever the line) (Figures 2(a) and 2(b)) and that the scale of such process is affected by the presence or absence of antisense* Sl-ERF.B.3* gene. This inhibition is significantly more enhanced in the presence of antisense* Sl-ERF.B.3* (antisense transgenic lines AS25 and AS38) than in its absence (untransformed WT lines) (Figure 2(c)). Considering stem height means, the* Sl-ERF.B.3* antisense tomato plants exhibited higher salt stress sensitivity. Consequently, we can emphasize that the presence of antisense* Sl-ERF.B.3* and the underexpression of* Sl-ERF.B.3* in transgenic plants contribute to the intensification of the NaCl negative effect on shoot elongation. Salt stress effect on plant growth inhibition is largely documented [36]. One explanation is that salt stress causes unavailable soil water, which leads to water deficit that contributes to stomata closure to minimize water loss, causing CO_2_ assimilation restriction, which influences adversely plant growth.

**Figure 3:**
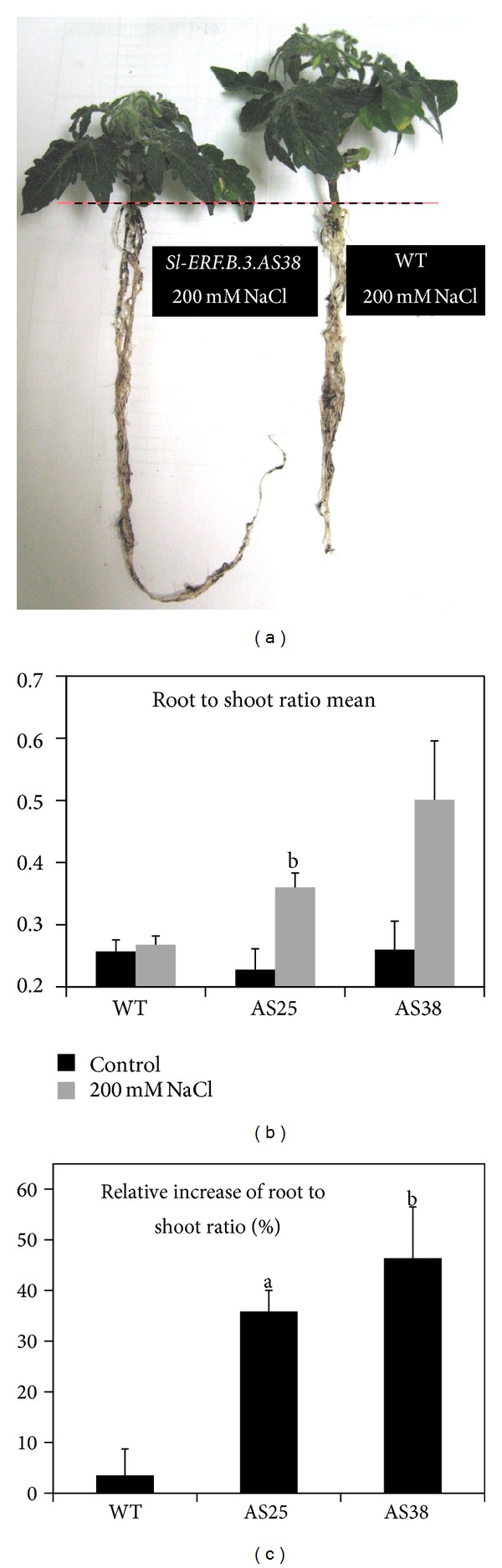
Salt stress effect on root to shoot ratio of* Sl-ERF.B.3* antisense transgenic lines (AS25 and AS38) and wild type (WT) tomato plants. (a) Photograph, taken at sampling time after six weeks of salt stress (regular irrigation by 200 mM NaCl solution), shows growth differences between wild type (WT) and* Sl-ERF.B.3*-antisense transgenic line (AS38). (b) Variation of root to shoot ratio mean under salt treatment (200 mM NaCl). (c) Relative increase rate of root to shoot ratio in response to sever salt stress treatment in wild type (WT) and* Sl-ERF.B.3* antisense transgenic lines (AS25 and AS38). Pairwise comparisons were made between wild type plants and antisense transgenic plants with Student's *t*-test (a: *P* < 0.05, b: *P* < 0.01). Data represent means and standard error of three replications.

As regards to the roots, salt stress application interestingly led to their elongation inhibition in WT but contributed to their stimulation in* Sl-ERF.B.3* antisense transgenic lines (Figures 2(d) and 2(e)). This salt stress dependent root elongation improvement reached up to 22% in AS38 line (Figure 2(e)). Our results clearly emphasize that the presence of tomato antisense* Sl-ERF.B.3* allows a significant stimulation of root elongation in response to salt stress. Taking into account the stem growth results, we can emphasize that under salinity and with comparison to WT, transgenic plants exhibited a differentially regulated growth, which is reduced in shoots but stimulated in roots (Figures 2 and 3(a)). Decrease of root to shoot ratio under salt stress was shown in several species as an important adaptive response.  Figure 3(b) shows clearly that 200 mM NaCl slightly affected the WT root to shoot rate (about 0.01 of increase corresponding to 3.5%) but induced its increase in AS25 line (about 0.13 of increase corresponding to 35%) and, nearly, its multiplication by two in AS38 line (Figure 3(c)).

**Figure 4:**
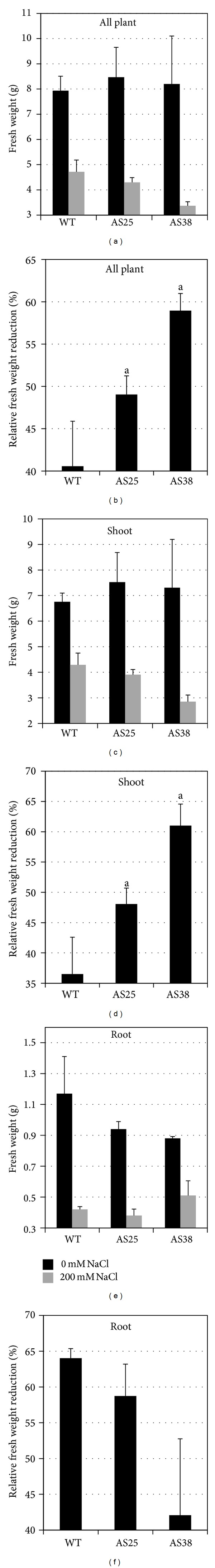
Salt stress effect on fresh biomass accumulation of* Sl-ERF.B.3* antisense transgenic lines (AS25 and AS38) and wild type (WT) tomato plants. Variation of fresh weight means under salt treatment (200 mM NaCl) and in control conditions (0 mM NaCl), in whole plants (a), in shoots (c), and in roots (e). Salt stress effect on relative fresh weight reduction is assessed in whole plants (b), in shoots (d), and in roots (f). Pairwise comparisons were made between wild type and transgenic plants with Student's *t*-test (a: *P* < 0.05, b: *P* < 0.01). Data represent means and standard error of three replications.

WT as well as the two* Sl-ERF.B.3* antisense transgenic lines displayed total fresh weight reduction, in response to salinity (Figure 4(a)). However, antisense plants were more affected by salt treatment. They displayed more pronounced total fresh weight reduction than that of WT (Figure 4(b)). Considering this growth parameter, salt stress-induced growth reduction is enhanced by the presence of antisense* Sl-ERF.B.3* gene. However, the monitoring of NaCl effect on fresh weight of different plant organs shows a clear discrimination between the aerial parts and roots. In comparison with WT, transgenic lines present the highest salt stress dependent fresh biomass reduction in shoots but the lowest one in roots (Figures 4(c), 4(d), 4(e), and 4(f)). The difference of relative fresh weight reduction values between transformed and untransformed plants reached up to more than 20% in root, especially for AS38 line (Figure 4(f)). Compared to WT plants, roots of transgenic plants were more protected from growth inhibition, generated by high NaCl concentration than shoots (Figures 4(d) and 4(f)). Plant growth is considered as coordinated biomass partitioning responses between shoots and roots. Growth of the shoot is more sensitive to salt stress than root growth [37]. Given that salinity results in the establishment of water deficit stress, longer root system should facilitate water absorption from deeper soil horizons and biomass increase. The differential response to salinity in both shoot and root is accompanied by differential changes in roots and shoots hormone concentrations. In particular, the inhibition of shoot growth in favor of a shift in biomass allocation to the root may be explained by a differential auxin to cytokinin ratio [37]. Root to shoot ratio is usually increased under salinity [38]. Root is the first plant organ encountering soil salinity, so the early salt stress response is first triggered in roots. In particular, after only 15 min of treatment, alteration of gene expression can be noted in roots, in particular, early response genes including transcriptional activators [39]. In this report we demonstrate that* Sl-ERF.B.3* is involved in the regulation of this adaptive response to salinity, because, in* Sl-ERF.B.3* antisense lines, shoots growth is much more sensitive to salt treatment than roots growth. Furthermore, it has been shown recently that root ethylene production is inhibited by salt treatment [40], because ethylene has an inhibitor role in root growth [37, 41]. The ethylene decrease in roots may protect roots from its growth inhibitor effect. For a large variety of plants, root growth is often less sensitive to salt stress than is growth of the shoot [42], including tomato plants [35].

### 3.3. Transgenic Plants Response to Cold Stress

**Figure 5:**
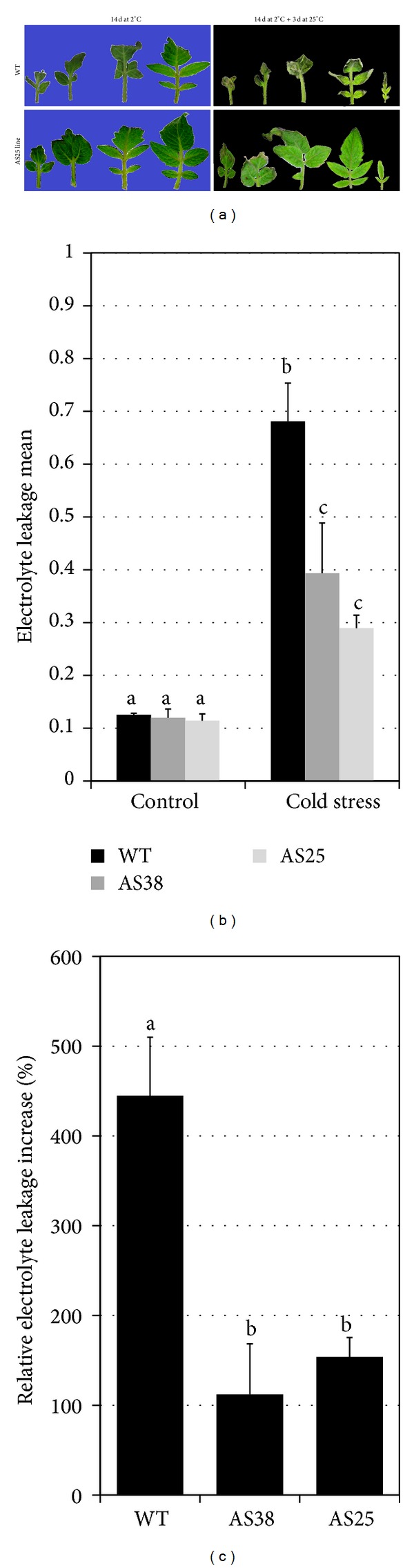
Cold stress effect on wild type and* Sl-ERF.B.3* antisense lines. (a) Photographs of both* Sl-ERF.B.3* antisense transgenic tomato lines (AS25) and wild type (WT) were taken after 14 days of cold treatment (2°C) then three days of recovery (25°C). Control: plants were grown under normal growth conditions. (b) Electrolyte leakage means are assessed in two* Sl-ERF.B.3* antisense transgenic tomato lines (AS25 and AS38) and in wild type (WT) treated or nontreated by incubation at 2°C temperature during 14 days. (c) Percentages of relative increase in electrolyte leakage between control (maintained at 25°C) and cold stressed (incubated at 2°C) plants of both wild type (WT) and* Sl-ERF.B.3* antisense transgenic lines (AS25 and AS38). Data are means of three replicates ± standard error. Same letters and different letters indicate, respectively, nonsignificant differences and significant differences between data, according to Student's *t*-test.

Response to cold stress was investigated in both WT and* Sl-ERF.B.3* transgenic plants, which exhibited different degrees of stress injury (Figure 5(a)). Tomato, a plant native to warm habitats, displays symptoms of injury upon low temperature exposure [43]. Ion leakage was measured to reflect the level of cellular damages after 14 d of cold treatment. Statistic analysis showed no significant differences between electrolyte leakage values assessed on all nonstressed plant tissues, whatever the line (Figure 5(b)). However, cold stress treatment data revealed significant differences between WT and transgenic lines (Figures 5(b) and 5(c)). Results, presented in Figure 5(b) showed that cold treatment leads to a strong increase in ion leakage in WT leaves tissue, reaching up 0.68, for only 0.28 and 0.39, respectively, in AS25 and AS38* Sl-ERF.B.3* antisense lines. This indicates that* Sl-ERF.B.3* antisense transgenic lines are able to maintain significantly, low percentage of electrolyte leakage increase in their leaf tissues, compared with WT and they are clearly more tolerant to cold stress. Thus, introducing antisense* Sl-ERF.B.3* into tomato plants may confer reduced plasma membrane injury and enhanced tolerance against low temperature stress. This conclusion is supported by the improvement of cold tolerance in the two independent transgenic lines expressing antisense* Sl-ERF.B.3, *with statistically no significant differences between measurements. Therefore,* Sl-ERF.B.3 *is a cold stress related gene, which may act as component of the cold stress response pathway in tomato, playing a role in the layout of cold stress symptoms. Recent study reported that the overexpression of an ERF transcription factor,* TaPIE1* in wheat reduces the plasma membrane damages, contributing to enhanced resistance to freezing, by upregulating a range of stress-related genes, downstream the ethylene signaling pathway [44].

### 3.4. *Sl-ERF.B.3* Sequence Study

**Figure 6:**
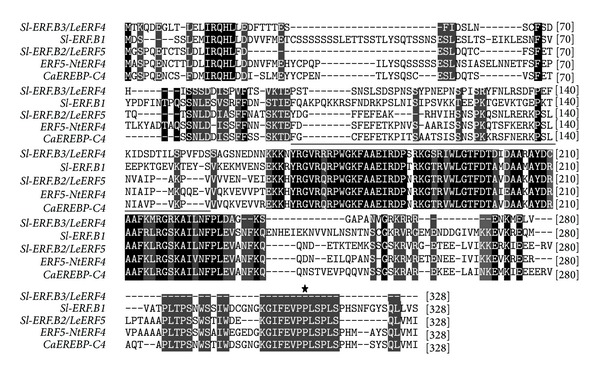
Multiple amino acid sequence alignment of* tomato Sl-ERF.B.3* with related amino acid sequences of* tomato Sl-ERF.B.1*,* tomato Sl-ERF.B.2*,* Capsicum annuum CaEREBP-C4* (AAX20037.1), and* Nicotiana tabacum NtERF4/ERF5* (Q40478.1). Conserved residues are shaded in black. Dark grey shading indicates similar residues in four out of five of the sequences. Alignments were made in ClustalW using the default parameters. The black bar above the sequences represents the ERF domain. Star represents putative MAP kinase phosphorylation site absent in* Sl-ERF.B.3* sequence. Dashes show gaps in the amino acid sequences introduced to optimize alignment. Numbers show the positions of amino acid residues.

To search for potential structural similarities between* Sl-ERF.B.3* and stress-involved proteins, we performed BLAST search using amino acid sequence of* Sl-ERF.B.3* and multiple amino-acid sequence alignment using formerly characterized stress related proteins. Data indicated that in addition to subclass B members (*Sl-ERF.B.1, Sl-ERF.B.2*),* Sl-ERF.B.3* exhibited sequence similarities with others ERF proteins that previous reports highlighted their involvement in plant stress responses,* CaEREBP-C4* and* ERF5/NtERF4. CaEREBP-C4* is a pepper transcription factor shown to be involved in cold stress responses [45], while* ERF5/NtERF4* was reported to be a transcriptional activator [46], involved in the early plant responses to wounding [47]. Until now,* Sl-ERF.B.3/LeERF4* has been only characterized as strongly induced against wounding stress [48]. In this study we demonstrated its involvement in tomato response to other abiotic stresses, including heat and cold stresses. At the perception level, both stresses affect the same site of temperature perception, the plasma membrane, but they trigger opposite changes in its fluidity, leading to the activation of different MAPK (mitogen-activated protein kinase) cascades [49]. Results, in Figure 6 show that* Sl-ERF.B.3* amino acid sequence lacks the putative MAP kinase phosphorylation site, found in C-terminal region of the two other members of subclass B and both of that of* NtERF4* and* CaEREBP-C4*. However,* Sl-ERF.B.3* includes in its amino acid sequence an acidic domain [48], which had been shown in other species to act as activation domain [50]. Considering the overall results, we can assert that* Sl-ERF.B.3* is a transcriptional activator that may probably be negatively involved in salt stress dependent growth regulation and in cold stress response. Further research should address this issue and allow better understanding of* Sl-ERF.B.3* role in cold and salt stress responses.

## Figures and Tables

**Table 1:** Primer sequences for expression study.

Name	Forward primer (5′→3′)	Reverse primer (5′→3′)
*Sl-ERF.B.3 *	CGGAGATAAGAGATCCAAGTCGAA	CTTAAACGCTGCACAATCATAAGC
*Sl-Actin-51 *	TGTCCCTATTTACGAGGGTTATGC	CAGTTAAATCACGACCAGCAAGAT
*CI7-254 *	GGCAATTTCATCTGAGTTGTCTGA	CTATTTGATCGATGAAGTTTCTTTTCC
*ACO1-694 *	AAGGGACTCCGCGCTCAT	AGTTGAAGGCCACTCACTTTGTC
*Hsp21-745 *	TTGGGTTTGATCCTTTCAGTTATG	ATAGCCATTTCTCTTCCTTCTGTTG
